# Research progress on mitochondria in bone defect repair: mechanisms and therapeutic implications

**DOI:** 10.3389/fbioe.2026.1763027

**Published:** 2026-02-05

**Authors:** Zhicheng Hu, Gang Chen, Zhisheng Long

**Affiliations:** 1 Jiangxi Medical College, Nanchang University, Nanchang, Jiangxi, China; 2 Jiangxi Provincial People’s Hospital, The First Affiliated Hospital of Nanchang Medical College, Nanchang, Jiangxi, China

**Keywords:** bone defect repair, mitochondria, mitochondrial dynamics, mitochondrial function, osteogenesis

## Abstract

Bone defect repair faces clinical challenges due to complex conditions caused by various factors such as trauma and aging. Traditional treatments have certain limitations, which seriously affect patients’ prognosis. As the core organelle of cells, mitochondria regulate the activity of key cells including osteoblasts, osteoclasts, and bone marrow mesenchymal stem cells through functions such as oxidative phosphorylation (OXPHOS), production and scavenging of reactive oxygen species (ROS), regulation of Ca^2+^ concentration, modulation of cell death, and immune response, as well as dynamic processes including fusion, fission, mitophagy, and transport. Moreover, mitochondria interact synergistically with the neuro-vascular-muscle axis, participating deeply in bone defect repair. This article systematically reviews the mechanisms and research progress of mitochondria in bone defect repair, providing a theoretical basis for the development of novel mitochondria-targeted repair strategies and facilitating the research and development of efficient clinical treatment regimens. This will help to develop new treatment strategies for bone defects. These strategies will be more effective, safe and targeted for individual patients.

## 1 Introduction

**FIGURE 1:**
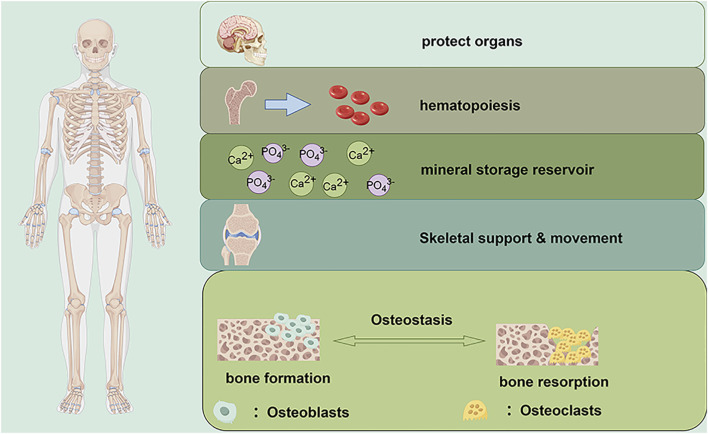
The Functions of Bone and the Mechanism of Bone Homeostasis. The functions include organs protection, hematopoiesis, mineral storage reservoir, skeletal support and movement. Under physiological conditions, osteoblasts and osteoclasts jointly maintain bone homeostasis.

Bone is a vital component of the human body, comprising various structures such as bone tissue, periosteum, and bone marrow (Clarke, 2008). Its functions are diverse (Figure 1), including providing support for the body and protection for vital organs, acting as a storage medium for calcium and phosphorus, and facilitating blood formation via red bone marrow (Sheehy et al., 2019). In healthy conditions, the internal environment of the bone is subject to a sophisticated regulatory mechanism, involving the guidance of new bone formation by osteoblasts and the absorption of old bone by osteoclasts, collectively maintaining bone homeostasis (Kim et al., 2020; Zhang et al., 2025). However, this balance can be influenced by various factors, including severe trauma, infection, bone tumour removal surgery, congenital bone malformations and osteoporosis (Zhang M. et al., 2022). These factors can disrupt bone homeostasis, leading to bone loss. The body possesses a natural repair system for bone defects, which maintains the integrity of bone structure and function through the coordinated action of mobilised cells and cytokines. But the capacity of bone to repair itself is limited by a “critical-sized defect (CSD)”, defined as the smallest bone defect that cannot be spontaneously healed by the body’s endogenous repair mechanisms without external intervention. When the defect volume is smaller than the CSD, the body can initiate self-repair through the process of endogenous repair without the need for external intervention (Mancuso et al., 2021). Whereas defects exceeding the CSD pose challenges for the endogenous repair system to achieve bone regeneration independently. In such instances, exogenous reconstructive measures such as autogenous bone grafting or biomaterial implantation become essential (Li et al., 2023). Otherwise, this will result in the failure of bone repair or poor quality of repair, which will have a significant impact on the patient’s ability to move and their quality of life. Despite the advent of a plethora of treatment modalities for bone defects, the complexity of the underlying pathologies, the heterogeneity of individual biological characteristics, and the limitations of existing treatments, the field of bone defect repair remains a significant clinical challenge.

Mitochondria, as highly dynamic and interconnected organelles (Mishra and Chan, 2016), are widely distributed throughout all eukaryotic cells except mature mammalian red blood cells. Serving as the cellular powerhouse, their core function is to convert nutrients into adenosine triphosphate (ATP) via oxidative phosphorylation (OXPHOS), thereby providing the energy necessary for cellular life processes. Moreover, mitochondria are deeply involved in numerous core cellular events, including reactive oxygen species (ROS) production and clearance, intracellular Ca^2+^ homeostasis regulation, cell death, and innate immune signalling activation (Bhatti et al., 2017). Thus, mitochondria play an irreplaceable core role in maintaining cellular homeostasis and regulating cell physiological functions by modulating osteoblastic/osteoclastic markers (Table 1). In recent years, with the continuous advancement of mitochondrial bioscience research, extensive basic and clinical studies have confirmed that disruption of mitochondrial structural integrity and functional abnormalities can impair osteocyte proliferation, differentiation, and functional activity, inducing or exacerbating a series of bone-related diseases (Yang et al., 2020), thereby significantly hindering the bone defect repair process. Given the critical regulatory role of mitochondrial functional homeostasis in bone tissue metabolism and repair, this paper aims to systematically review research progress on mitochondrial regulation of bone defect repair, explore potential mechanisms of action, and discuss future application potential.

**TABLE 1:** Common osteoblastic and osteoclastic markers.

Gene	Protein	Main function and molecular mechanism	Phase	Core signaling pathway
BMP2	Bone morphogenetic protein 2	Secreted ligand; Smad1/5/8-P → RUNX2 transcription	Osteo-induction	BMP-Smad
WNT1	Wingless-type MMTV integration site family, member 1	Ligand; stabilises β-catenin → TCF/LEF targets	Commitment and mechano-response	Canonical Wnt/β-catenin
RUNX2	Runt-related transcription factor 2	Master TF; heterodimer with CBFβ → osteoblast gene battery	Early commitment	BMP-Smad, Wnt/β-catenin
SP7	Osterix	Zinc-finger TF; downstream of RUNX2, represses chondrogenesis	Mid differentiation	BMP-Smad, Notch
COL1A1	Collagen α-1(I) chain	Scaffold for mineral deposition; binds integrins	Matrix formation	TGF-β/integrin-FAK
ALPL	Tissue-nonspecific alkaline phosphatase	Hydrolyses PPi → Pi; promotes hydroxyapatite nucleation	Mineralisation	Wnt/β-catenin (indirect)
RANKL	Receptor activator of NF-κB ligand	Trimerises RANK; master driver of osteoclast differentiation	Differentiation and activation	RANKL-RANK-OPG
RANK	Tumor necrosis factor receptor superfamily member 11A	RANKL receptor; TRAF6 → NF-κB and NFATc1	Commitment	RANKL-RANK-OPG
CTSK	Cathepsin K	Cysteine protease; cleaves type I collagen at low pH	Matrix resorption	SRC-AKT-NF-κB
MMP9	Matrix metalloproteinase-9	Gelatinase; degrades denatured collagen, releases TGF-β	Resorption and angiogenic coupling	MAPK-JNK/PI3K-Akt
ACP5	Tartrate-resistant acid phosphatase	Dephosphorylates matrix proteins; generates ROS to amplify resorption pit	Maturation and function	SRC-RAC1-NOX
OPG	Osteoprotegerin	Decoy receptor; neutralises RANKL.	Resorption control	RANKL-RANK-OPG

## 2 Functions of mitochondria

### 2.1 Oxidative phosphorylation

**FIGURE 2:**
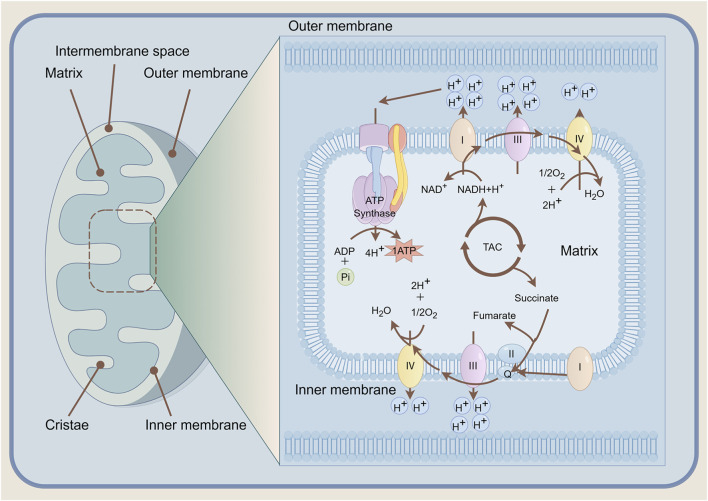
Mitochondrial Architecture and the Mechanism of Oxidative Phosphorylation. NADH/FADH_2_ donate electrons to the inner-membrane respiratory chain (complexes I–IV), coupling redox energy to proton pumping; the generated H^+^ electrochemical gradient powers ATP synthase to phosphorylate ADP. Molecular oxygen serves as the terminal electron acceptor, forming water and sustaining electron flow. NADH nicotinamide adenine dinucleotide, FADH_2_ flavin adenine dinucleotide, ATP adenosine triphosphate, ADP adenosine diphosphate. TAC tricarboxylic acid cycle.

In recent years, extensive research has demonstrated that disturbances in energy metabolism—such as abnormal metabolism of glucose, amino acids, and lipids—disrupt bone homeostasis, triggering or exacerbating bone loss and significantly increasing the risk of osteoporotic fractures. Mitochondria, serving as the primary sites for cellular biooxidation and energy conversion, efficiently synthesise ATP to power all physiological activities, earning them the widespread designation as the ‘powerhouses’ of the cell (Figure 2). Cellular energy metabolism primarily relies on two interconnected pathways (Rigoulet et al., 2020): the anaerobic process of glycolysis, and the aerobic process of oxidative phosphorylation. However, oxidative phosphorylation exhibits a marked advantage in energy production efficiency compared to glycolysis (Dobson et al., 2020). This disparity renders Bone Marrow mesenchymal Stem Cells (BMSCs) more reliant on oxidative phosphorylation to meet their energy demands during the osteogenic differentiation stage.

BMSCs are adult stem cells of mesodermal origin, possessing self-renewal capacity and multipotent differentiation potential. Under specific induction conditions, they can be directed to differentiate into mesoderm-derived cell lineages such as osteoblasts, chondrocytes, and adipocytes (Li et al., 2017; Han et al., 2019). Notably, a characteristic ‘differentiation equilibrium’ exists between osteogenic and adipogenic differentiation in BMSCs (Hsu et al., 2016), wherein enhanced differentiation capacity in one pathway typically accompanies diminished capacity in the other. This imbalance constitutes a key mechanism underlying diseases such as osteoporosis. Mitochondrial function and oxidative phosphorylation activity represent central regulators of this equilibrium, with relevant studies providing partial evidence. Li B. et al. (2022) discovered that nicotinamide adenine dinucleotide (NAD^+^) positively regulates osteogenic differentiation in BMSCs by modulating the mitochondrial OXPHOS pathway. Conversely, treatment with the NAD^+^ inhibitor FK866 significantly downregulated the expression of osteogenesis-related genes, ultimately reducing new bone formation at bone defect sites in animal models. Moreover, mitochondrial morphological and functional remodelling is intrinsically linked to osteogenic differentiation. Studies indicate that during BMSCs differentiation towards osteocytes or adipocytes, the mitochondrial-to-cytoplasmic area ratio increases (Forni et al., 2016), whilst individual mitochondrial surface area remains largely stable. This implies a marked increase in mitochondrial number during differentiation (Li et al., 2017), directly facilitating the assembly of oxidative phosphorylation complexes and enhancing cellular OXPHOS activity and ATP production capacity. Mitochondrial dysfunction inhibits osteogenic differentiation: Research by He et al. (2021) confirmed that defects in the mitochondrial tRNA modifying enzyme Mtu1 result in insufficient 2-thiouridine modification of mitochondrial tRNAs. This leads to a marked reduction in the activity of mitochondrial respiratory chain complexes I, III, and IV, impairing OXPHOS function. This mitochondrial dysfunction inhibits the osteogenic differentiation potential of BMSCs, ultimately resulting in an osteoporotic phenotype in mice (reduced bone density/disrupted trabecular structure). Given the positive regulatory role of mitochondrial function in osteogenic differentiation, targeting mitochondrial OXPHOS activity has emerged as a potential strategy for promoting bone repair. Guo et al. (2020) developed a mitochondrial transplantation-based intervention: introducing healthy mitochondria into BMSCs significantly upregulated cellular OXPHOS activity and markedly increased ATP production. Further investigations revealed that mitochondrial transplantation not only enhances the *in vitro* proliferation and migration capabilities of BMSCs but also elevates their osteogenic potential by activating osteogenic differentiation pathways (such as the Wnt/β-catenin pathway). Moreover, it effectively promotes *in situ* bone repair in animal bone defect models. This research provides experimental evidence for mitochondrial-targeted therapies in bone metabolic disorders. Notably, oxygen and nutrient supply within the microenvironment constitute prerequisites for mitochondrial OXPHOS function. Under physiological conditions, functional osteoblasts responsible for bone matrix synthesis predominantly reside in perivascular regions. This anatomical arrangement ensures adequate oxygen and metabolic substrate availability for osteoblasts, establishing favourable conditions for oxidative phosphorylation. Further studies by Van Gastel and Carmeliet (2021) confirmed that enhanced OXPHOS activity synchronously elevates the expression levels of antioxidant enzymes (such as SOD) within BMSCs, significantly bolstering cellular antioxidant stress resistance. Conversely, inhibiting OXPHOS activity via chemical inhibitors (such as antimycin A) or inducing oxidative stress with exogenous hydrogen peroxide impedes osteogenic differentiation in BMSCs, manifested by downregulated osteogenic gene expression and reduced mineralised nodule formation. This finding reveals the synergistic role of OXPHOS activity and cellular redox homeostasis in osteogenic differentiation.

### 2.2 The generation and clearance of ROS

**FIGURE 3:**
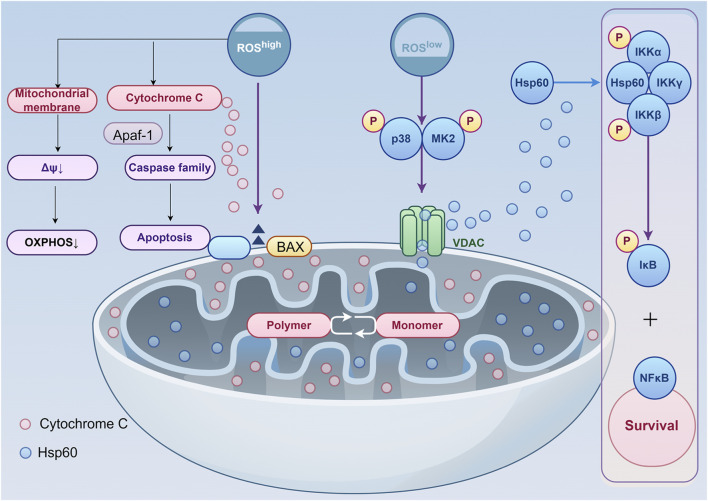
The Concentration-Dependent Dual Role of ROS in Regulating Mitochondria-Mediated Apoptosis and Cell Survival. At low levels, ROS act as signaling molecules that activate the p38-MK2-Hsp60 axis, promoting NF-κB mediated pro-survival gene expression and maintaining mitochondrial homeostasis. In contrast, elevated ROS trigger apoptosis through BAX oligomerization, cytochrome c release, and apoptosome formation, while concurrently inducing mitochondrial membrane potential collapse and impairing oxidative phosphorylation. ROS reactive oxygen species, ΔΨ mitochondrial membrane potential, OXPHOS oxidative phosphorylation, Apaf-1 apoptotic protease activating factor 1, Caspase family cysteine aspartate specific protease family, BAX BCL2-associated X protein, VDAC voltage-dependent anion channel, Hsp60 heat shock protein 60, p38 p38 mitogen-activated protein kinase, MK2 mapk-activated protein kinase 2, IKKα/IKKγ/IKKβ IκB kinase α/γ/β, IκB inhibitor of NF-κB, NF-κB nuclear factor kappa-light-chain-enhancer of activated B cells.

ROS constitute a class of highly reactive oxygen-derived small molecules, encompassing both free radicals such as superoxide anion (O_2_^−^) and hydroxyl radical (•OH), as well as non-radical species including hydrogen peroxide (H_2_O_2_) and singlet oxygen (^1^O_2_). Mitochondria serve as the primary site for ROS generation, where both free radicals and non-radicals are continuously produced and converted (Liu et al., 2024). ROS exhibit dual effects (Figure 3): at physiological levels, they function as crucial intracellular molecular signals, participating in the regulation of cellular processes such as proliferation, differentiation, and apoptosis (Sart et al., 2015); conversely, when ROS levels exceed the cell’s scavenging capacity, they can directly damage cellular structure and function by attacking cell membranes (Tsang et al., 2014). To counteract potential ROS damage, mitochondria rely on a sophisticated antioxidant defence system to maintain homeostasis (Ighodaro and Akinloye, 2018). This comprises both enzymatic antioxidants, such as SOD and CAT, and non-enzymatic antioxidants, including glutathione (GSH) and vitamin C. These work synergistically to scavenge excess ROS, thereby safeguarding cellular survival and function. Therefore, maintaining cellular ROS homeostasis is of critical importance for bone defect repair.

When the body’s oxidative and antioxidant systems become imbalanced, excessive ROS can induce oxidative stress. Oxidative stress not only induces osteoblast apoptosis but also promotes osteoclast formation (Tao et al., 2020; Zan et al., 2023), ultimately impeding bone defect repair. Existing research has confirmed that multiple strategies regulating ROS homeostasis can improve bone repair outcomes. Research by Qiu et al. (2019) revealed that melatonin regulates ROS levels through a dual mechanism. On one hand, it upregulates the expression of antioxidant enzymes such as SOD and CAT, enhancing ROS scavenging capacity. On the other hand, it downregulates the expression of NADPH oxidases (NOX1 and NOX2), reducing ROS sources, while further diminishing ROS production by inhibiting the NF-κB pathway. Moreover, melatonin antagonises TNF-α induced excessive ROS production. TNF-α itself inhibits osteoblast differentiation by activating NF-κB and MAPK pathways whilst suppressing the SMAD1/5/8 pathway (Zuo et al., 2018). Thus, melatonin promotes BMSCs osteogenic differentiation via this pathway (Qiu et al., 2019). Notably, physiologically concentrated ROS exert positive regulatory effects on bone repair: studies by Khalid et al. (2020) demonstrated that low-concentration ROS induced by extracellular phosphate (Pi) and H_2_O_2_ activate the ERK1/2 pathway, upregulating the expression of osteogenic markers such as osteopontin (OPN), thereby promoting extracellular matrix mineralisation. Furthermore, interventions targeting oxidative stress regulation demonstrate bone repair potential: Zheng et al. (2020) confirmed that 4-octyl itaconic acid ester (4-OI) enhances osteoblast antioxidant capacity by activating the Nrf2 signalling pathway, thereby suppressing H_2_O_2_ induced oxidative damage and osteoblast death. Within materials science, ROS responsive bone defect repair materials designed by Sun H. et al. (2023) and Liu et al. (2024) release active components in response to the high ROS microenvironment within bone defects. This simultaneously improves local redox balance while synergistically promoting osteogenic differentiation and angiogenesis, and inhibiting osteoclast activity, thereby effectively enhancing bone defect repair efficiency.

### 2.3 Regulation of Ca^2+^ concentration

The chemical composition of bone is primarily divided into organic and inorganic components, which synergistically confer the biomechanical properties of flexibility and strength upon the skeletal structure. The organic component consists mainly of collagen fibres and an amorphous matrix, whose reticular structure provides the bone with resilient and elastic support. The inorganic components, also termed bone salts, comprise ions such as calcium and phosphorus. These are deposited within the organic matrix in the crystalline form of hydroxyapatite, forming the material foundation for bone strength and compressive resistance. When bone salts are deposited rhythmically within osteoid, the latter progressively transforms into hard bone tissue—a process termed bone calcification. The generation and deposition of bone salts depend upon the regulated activity of osteoblasts, which primarily originate from the directed differentiation of BMSCs. Both the differentiation of BMSCs into osteoblasts and the subsequent mineralisation process are precisely regulated by ROS, with this regulation relying on the synergistic interaction between osteogenic induction signals and mitochondrial function. Osteogenic induction regulates ROS production via two pathways: firstly, by enhancing mitochondrial activity to promote ROS generation from the respiratory chain; secondly, by activating the BMP-2 signalling pathway (Li Y. et al., 2022), which upregulates NADPH oxidase activity to further increase ROS production. In mature osteoblasts, physiological levels of ROS are generated in response to Pi stimulation, thereby activating extracellular signal-regulated kinase ERK1/2 and OPN expression (Khalid et al., 2020), ultimately promoting extracellular matrix mineralisation.

Mitochondrial regulation of Ca^2+^ homeostasis participates in bone mineralisation, and thus Ca^2+^ imbalance significantly impedes bone repair processes (Shuai et al., 2021; Van Gastel and Carmeliet, 2021). The mitochondrial calcium uniporter (MCU), a specific Ca^2+^ channel located in the inner mitochondrial membrane, primarily functions to transport Ca^2+^ from the cytoplasm into the mitochondrial matrix along its concentration gradient. By regulating mitochondrial Ca^2+^ levels, the MCU indirectly participates in numerous physiological processes including cellular Ca^2+^ homeostasis, energy metabolism, apoptosis pathway regulation, and sarcolemmal repair (D’Angelo and Rizzuto, 2023). Consequently, it may modulate osteoblastic mineralisation function by influencing Ca^2+^ signalling and energy supply within osteoblasts. Abnormal Ca^2+^ homeostasis constitutes a common pathological basis for multiple diseases (Modesti et al., 2021). In bone defects, excessive mitochondrial Ca^2+^ uptake triggers a cascade of reactions. On one hand, excessive Ca^2+^ accumulation disrupts the mitochondrial membrane potential, inhibiting oxidative phosphorylation and reducing ATP synthesis (Antony et al., 2016), thereby impairing osteoblast function. Concurrently, abnormal mitochondrial Ca^2+^ uptake promotes electron leakage in the respiratory chain, resulting in substantial ROS production (Chen et al., 2024). Excessive ROS not only damages mitochondrial structure (establishing a vicious cycle of “mitochondrial injury–increased ROS”), but also oxidatively modifies bone matrix proteins, thereby suppressing osteoblast activity and promoting apoptosis. This ultimately exacerbates the impairment of bone defect repair.

### 2.4 Cell death

Cell death represents the terminal stage of the cellular life cycle. As the pivotal regulatory hub of apoptosis, the functional state of mitochondria directly influences the progression of bone defect repair. Upon initiation of the apoptotic programme, mitochondria undergo a series of characteristic alterations (Saunders et al., 2024), the most critical being the release of cytochrome c (cyt c), regarded as the core element in mitochondrial regulation of apoptosis (Glover et al., 2024). This release activates the caspase cascade, ultimately inducing cell death. Bao et al. (2024) demonstrated that interferon-γ and TNF-α directly induce osteoblast death by downregulating the anti-apoptotic protein B-cell lymphoma 2 (Bcl-2) and promoting mitochondrial cyt C release. Furthermore, as a key signalling molecule in apoptosis, elevated ROS levels form a vicious cycle with mitochondrial dysfunction: increased ROS production during apoptosis leads to mitochondrial membrane potential decline or collapse, enhancing inner mitochondrial membrane permeability and further promoting cyt C release (Morse et al., 2024). These mechanisms demonstrate that ROS and mitochondria interact to regulate apoptosis in bone tissue cells, directly influencing the efficiency of bone defect repair.

Iron is an essential trace element for maintaining osteoblast differentiation and mineralisation, yet excessive iron accumulation can disrupt skeletal homeostasis by inducing ferroptosis. The essence of ferroptosis lies in the imbalance of the cellular antioxidant system: under normal physiological conditions, Glutathione Peroxidase 4 (GPX4) relies on GSH to scavenge excess ROS, thereby maintaining redox equilibrium. However, during iron overload, GPX4 activity markedly diminishes and GSH becomes depleted. Intracellular Fe^2+^ then catalyse lipid peroxidation via the Fenton reaction, generating substantial ROS. This ultimately leads to cell membrane damage, mitochondrial dysfunction, and cell death (Tang et al., 2021; Yan et al., 2021). Consequently, disruption of bone homeostasis constitutes a significant precipitating factor in abnormal bone metabolism.

With advancing age, iron gradually accumulates within the body alongside elevated ROS levels. This disrupts bone homeostasis through a dual mechanism: inhibiting osteoblast function while promoting osteoclast activity, thereby increasing the risk of osteoporosis. Borriello et al. (2016) observed that although iron overload promotes proliferation of BMSCs and accelerates their entry into the S phase of the cell cycle, it simultaneously suppresses their differentiation into osteoblasts and extracellular matrix calcification, directly impairing osteogenic capacity. Che et al. (2021) confirmed that iron overload induces osteoblast apoptosis by elevating ROS levels, disrupting calcium homeostasis, and subsequently triggering mitochondrial dysfunction through activation of the p-eIF2α/ATF4/CHOP pathway. Furthermore, Xia et al. (2023) discovered that *REPIN1* gene knockout mitigates iron overload toxicity in osteoblasts by downregulating LCN2 expression, modulating protein levels of Bcl-2, cyt c, and BAX in the mitochondrial apoptosis pathway, thereby indirectly preserving osteoblast viability. Osteoclasts are the sole cells *in vivo* capable of bone resorption. Iron overload enhances their activity through multiple mechanisms: iron overload, Fe^2+^ within osteoclasts generates substantial ROS via the Fenton reaction, activating the MAPK signalling pathway and directly promoting bone resorption (Chen Y. et al., 2023). Guo et al. (2024) further demonstrated that iron overload promotes osteocyte apoptosis via oxidative stress and upregulates receptor activator of nuclear factor κB ligand (RANKL) expression. As a key factor in osteoclast differentiation, RANKL significantly enhances osteoclast maturation and bone resorption activity.

### 2.5 Immune response

**FIGURE 4:**
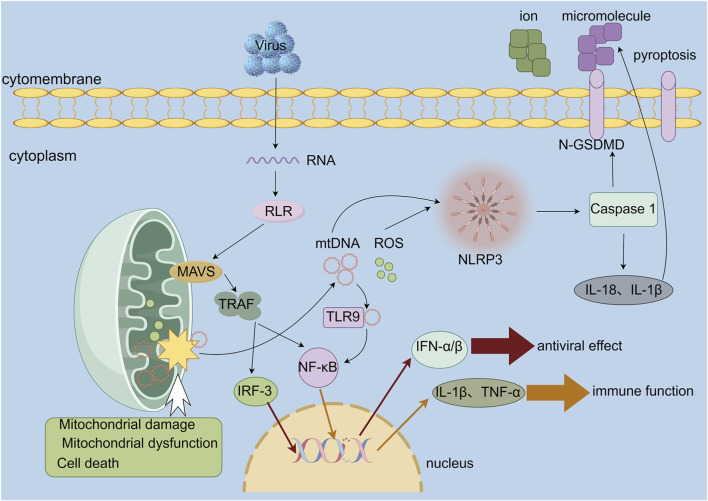
Mitochondrial Stress as a Central Hub for Initiating Diverse Immune Signaling Pathways. The RLR-MAVS pathway activates transcription factors (IRF3, NF-κB) to induce interferons and pro-inflammatory cytokines. Simultaneously, mitochondrial damage releases mtDNA/ROS, activating the NLRP3 inflammasome. Active caspase-1 cleaves GSDMD to trigger pyroptosis and processes IL-1β/IL-18 for secretion, amplifying immune responses. RLR RIG-I-like receptors, MAVS mitochondrial antiviral signaling protein, TRAF tumor necrosis factor receptor-associated factor, IRF-3 interferon regulatory factor 3, mtDNA mitochondrial DNA, TLR9 toll-like receptor 9, NLRP3 nod-like receptor pyrin domain-containing protein 3, GSDMD gasdermin d, IFN-α/β interferon α/β, IL-1β/18 interleukin-1β/18, TNF-α tumor necrosis factor α.

Mitochondria are not only a source of energy for the cell, but also a crucial regulator of the immune response. The core function is to release damage-associated molecular patterns (DAMPs) to initiate the immune response (Figure 4). When mitochondria are damaged or the cell dies, the mitochondria DNA (mtDNA) and ROS are released into the cytoplasm. These act as DAMPs to activate several innate immune pathways, including the MAVS, cGAS-STING, and NLRP inflammasome pathways (Zhong et al., 2018), which in turn trigger inflammatory responses. This process serves as both a ‘warning signal’ for cell damage (Mills et al., 2017) and contributes to the development of inflammatory responses, providing a new direction for targeted therapy for immune-related bone loss. *Staphylococcus aureus* (*S. aureus*) is the most prevalent causative agent of bacterial infections in the musculoskeletal system (Ma et al., 2024). It is also the primary cause of bone infections. *Staphylococcus aureus* disrupts the process of bone repair through multiple mechanisms that cause cell death in bone-forming cells and stimulate the differentiation of osteoclasts and the expression of bone-resorbing factors, thereby accelerating bone dissolution (Li et al., 2020). Ultimately, this delays bone healing. In the infected state, maintaining the stability of the mitochondria is crucial for preventing the spread of inflammation and protecting the function of bone cells. In recent years, new breakthroughs have been made in the field of targeted mitochondria immune regulation for bone repair: Ghosh et al. (2022) designed VCD-077, which carries bone cement, and achieved the dual goals of preventing infection and repairing bone defects through the “drug-device” synergistic effect. Chen X. et al. (2023) constructed the chameleon-like HA@Ce-TA nanoplatform, which not only remodels mitochondrial dynamics by upregulating mitochondrial fusion genes but also mimics SOD and CAT activity to scavenge excess ROS. This achieves synergistic antibacterial and anti-inflammatory effects, effectively enhancing bone repair efficiency within the infected microenvironment.

In addition, BMSCs secrete cell-derived extracellular vesicles (EVs), which are rich in functional nucleic acids, such as microRNA and tRNA. These EVs have a unique advantage in promoting bone repair by regulating bone differentiation, vascularisation and inflammation (Zhang M. et al., 2022). Furthermore, Liu et al. (2020) have demonstrated that, under hypoxic conditions, HIF-1α mediated EVs secretion is increased, which significantly enhances the bone-forming and vascularisation abilities of EVs derived miR-126. Consequently, combining the regulation of the immune microenvironment with the EVs secretion of BMSCs is expected to provide a multifaceted regulatory mechanism that can “modify the immune microenvironment - promote bone regeneration” and provide a more effective treatment for infected bone defects.

## 3 Mitochondrial dynamics

**FIGURE 5:**
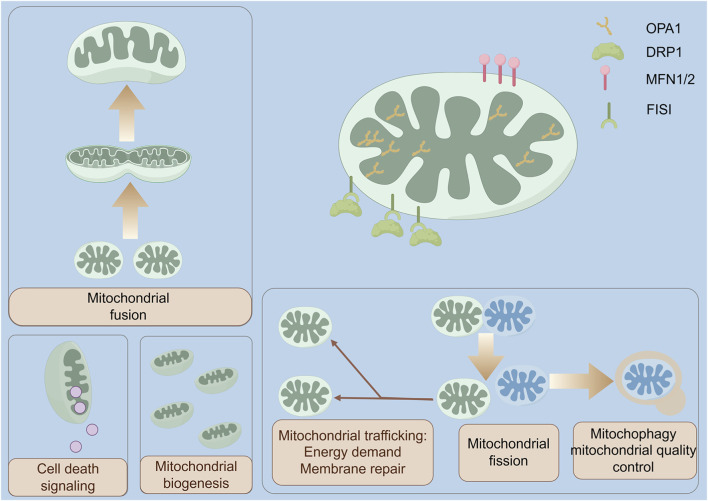
Integrated Overview of Mitochondrial Dynamics, Quality Control, and Signaling Functions. Mitochondrial fusion requires outer-membrane mitofusins (MFN1/2) and inner-membrane OPA1 to merge lipid bilayers and cristae, whereas cytolic DRP1 is recruited by FIS1 to constrict and divide the organelle, thereby regulating network morphology, quality control, and apoptosis signaling. DRP1 dynamin-related protein 1, MFN1/2 mitofusin 1/2, FIS1 mitochondrial fission 1 protein, OPA1 optic atrophy 1.

Mitochondrial dynamics encompasses the comprehensive biological processes describing mitochondrial structure and function, primarily involving the coordinated processes of mitochondrial fusion and fission, biosynthesis, autophagy, and transport (Altieri, 2019; Dorn, 2019). These processes, through precise regulation, collectively maintain mitochondrial morphological plasticity, functional integrity, and quantitative homeostasis (Figure 5). When mitochondrial dynamics become imbalanced, mitochondrial structural assembly and functional execution are disrupted, thereby forming a crucial pathological basis for various diseases, including neurodegenerative disorders, cardiovascular diseases, cancer, and bone-related conditions such as osteoporosis (Chan, 2020). Therefore, elucidating the molecular regulatory mechanisms of mitochondrial dynamics and clarifying the roles of key dynamic proteins in disease progression may provide novel therapeutic targets for the targeted treatment of these conditions (Zacharioudakis and Gavathiotis, 2023).

### 3.1 Mitochondrial fusion

Mitochondrial fusion denotes the process whereby two or more mitochondria merge their membranes to form larger mitochondria or a network of interconnected mitochondria. With advancing age, cellular senescence leads to the accumulation of mtDNA mutations. Mitochondrial fusion maintains mitochondrial function and genetic stability by facilitating material exchange and information communication between distinct mitochondria (Pernas and Scorrano, 2016). Mitochondrial fusion requires coordinated action between the outer and inner mitochondrial membranes, regulated by specific proteins: Mitochondrial Fusion Proteins 1/2 (MFN1/2) govern outer membrane fusion, while Optic Atrophy Protein 1 (OPA1) governs inner membrane fusion. The synergistic action of these proteins maintains the dynamic equilibrium of the mitochondrial network, safeguarding mtDNA integrity and ensuring efficient respiratory chain function.

Recent studies have confirmed that mitochondrial fusion directly influences bone homeostasis and defect repair by regulating osteocyte metabolism and differentiation. Yue et al. (2014) discovered that the diterpene derivative 15-oxospirolaminolactone (S3) enhances non-degradative ubiquitination of MFN1/2 by inhibiting the deubiquitinating enzyme USP30, thereby restoring normal mtDNA distribution, mitochondrial membrane potential, and oxidative phosphorylation function. While enhanced MFN1/2 activity promoted mitochondrial fusion, providing an energy foundation for osteogenic differentiation. Fan et al. (2020) found that miR-181c enhances mitochondrial fusion by regulating the AMPK-MFN1 signalling pathway, effectively mitigating oxidative stress-induced damage to BMSCs and offering a novel therapeutic target for craniofacial bone defect reconstruction. Furthermore, Huo et al. (2022) demonstrated that MFN1/2 overexpression induces mitochondrial clustering and peroxidase enrichment, wherein peroxidase scavenges excess ROS and alleviates oxidative stress, potentially promoting bone defect repair. Concerning osteoclasts, Ballard et al. (2020) discovered that MFN2 deficiency inhibits the *in vitro* differentiation capacity of osteoclast precursors and reduces their sensitivity to RANKL-induced bone resorption, suggesting MFN2 is indispensable for osteoclast maturation and functional maintenance.

### 3.2 Mitochondrial fission

Mitochondrial fission refers to the process whereby a single mitochondrion divides into two smaller daughter mitochondria. This process is crucial for maintaining mtDNA integrity and membrane potential stability. Mitochondrial fission enables the segregation of segments carrying mutated mtDNA and depolarised membrane structures into specific daughter mitochondria. Abnormal mitochondria are subsequently cleared via the ubiquitin-proteasome system or mitochondrial autophagy, thereby preserving mitochondrial functionality (Quintana-Cabrera and Scorrano, 2023). The core regulatory molecule for mitochondrial fission is dynamin-related protein 1 (DRP1). Beyond mediating fission, it also participates in regulating osteoclast differentiation by influencing the formation of actin rings on the osteoclast surface.

Mitochondrial fission is crucial for maintaining the stem cell characteristics of BMSCs. Inhibition of this process leads to reduced expression of BMSC-specific surface markers and significantly diminishes their multipotent differentiation potential (Ren et al., 2020). Research by Wang et al. (2024b) revealed that high-salinity environments induce upregulation of DRP1 expression, leading to excessive mitochondrial fission that disrupts normal mitochondrial morphology and function. This inhibits activation of the canonical Wnt signalling pathway, significantly impairing the osteogenic differentiation capacity of BMSCs and ultimately resulting in bone loss in mice. The study further revealed that exosomes can restore the osteogenic capacity of damaged BMSCs by transferring healthy mtDNA, offering a novel approach for bone regeneration. Abnormal mitochondrial fission is closely associated with tissue calcification, and multiple studies confirm that targeted inhibition of DRP1 can improve calcification pathology. Chen et al. (2020) demonstrated that melatonin suppresses vascular calcification by inhibiting mitochondrial fission through activation of the AMPK/DRP1 signalling pathway. Rogers et al. (2017) similarly demonstrated that DRP1 inhibition reduces vascular calcification under conditions of collagen secretion and oxidative stress. These studies collectively indicate that DRP1 inhibition effectively mitigates vascular calcification. As a key target regulating calcification, DRP1 inhibitors or modulators hold potential for improving vascular calcification or abnormal bone matrix calcification that may accompany bone defect repair.

### 3.3 Mitochondrial autophagy

Mitochondrial autophagy is a receptor-mediated selective autophagy process whose core function is the targeted clearance of damaged or dysfunctional mitochondria in response to metabolic stresses such as inflammation, hypoxia, genotoxicity, and malnutrition (Wang et al., 2020). Impairment of mitochondrial autophagy leads to substantial accumulation of damaged mitochondria and ROS, subsequently inhibiting osteogenesis and exacerbating bone resorption (Wang et al., 2023; Pays, 2025). Thus, maintaining mitochondrial homeostasis and cellular energy metabolism equilibrium through mitochondrial autophagy creates favourable conditions for bone defect repair.

The functional activity of BMSCs is intrinsically linked to autophagy, primarily regulating bone tissue repair and regeneration via two mechanisms (Ceccariglia et al., 2020): On one hand, autophagy safeguards mitochondrial oxidative phosphorylation by eliminating damaged mitochondria and excess ROS, thereby promoting the directed differentiation of BMSCs into osteoblasts. On the other hand, BMSCs modulate the autophagic capacity of immune cells, mitigating local inflammatory responses and furnishing a suitable microenvironment for bone regeneration. Research by An et al. (2018) indicates that autophagy regulates the paracrine function of BMSCs. Following autophagy enhancement via rapamycin or inhibition via si-Beclin1, it was observed that increased autophagy significantly elevated BMSCs secretion of vascular endothelial growth factor (VEGF). VEGF, in turn, promotes angiogenesis by activating the MAPK/ERK signalling pathway, thereby indirectly supplying nutrients and oxygen to the bone defect site and aiding the bone repair process.

As the core cells of bone formation, the survival and function of osteoblasts depend on the precise regulation of mitochondrial autophagy. Research indicates that autophagy levels are significantly upregulated during osteoblast differentiation. Appropriate levels of autophagy maintain mitochondrial functional homeostasis, thereby safeguarding osteoblast survival and core functions such as bone matrix synthesis and mineralisation (Wang et al., 2023). Mitochondrial autophagy positively regulates osteoblast proliferation and differentiation through multiple signalling pathways including SIRT1, PINK1, FOXO3, and PI3K (Wang et al., 2020). Furthermore, inhibiting autophagy elevates intracellular oxidative stress levels, not only suppressing osteoblast differentiation but also inducing apoptosis. Conversely, promoting autophagy effectively scavenges ROS and rescues cells from apoptosis, thereby preserving osteoblast activity (Yang et al., 2014).

### 3.4 Mitochondrial transport

Mitochondrial transport refers to the biological process whereby mitochondria move directionally within a single cell along the cytoskeleton, or transfer between different cells via intercellular contacts, secretion, and other mechanisms (Brestoff et al., 2025). This process is central to the cell’s allocation of mitochondria according to local energy demands and maintenance of regional functional homeostasis. Intracellular mitochondrial transport primarily relies on the microtubule system as its ‘track’, coordinated by a series of proteins: transport-associated proteins such as TRAK1 and KIF5B bind to mitochondrial Rho GTPase 1 (MIRO1) on the mitochondrial membrane, forming functional complexes that guide mitochondria along microtubules in a directed manner (Ding et al., 2025).

MIRO1 is a core protein in the mitochondrial transport mechanism and an essential molecule mediating mitochondrial transport (Castro et al., 2018). In the study by Liao et al. (2024): conditional knockout of the *Rhot1* gene encoding MIRO1 in osteoblasts revealed significant reductions in mitochondrial number, translocation rate, the area ratio of mitochondria to trans-cortical vessels (TCV), and the number of TCV branches. Furthermore,the study confirmed that vascular endothelial cells acquiring mitochondria from osteoblasts effectively restored endothelial dysfunction. This manifested as enhanced OXPHOS activity, reduced ROS production, and improved angiogenic capacity, ultimately promoting healing of cortical bone defects. These findings indicate that MIRO1-mediated mitochondrial transfer from osteoblasts to vascular endothelial cells plays a crucial role in maintaining TCV vascularisation and establishing the blood supply microenvironment essential for bone repair, demonstrating potential value in promoting bone regeneration.

Recent studies have demonstrated that mitochondrial transfer occurs among osteoblasts, osteoclasts, and BMSCs. This process is mediated by either cell-cell contact or exosomes, and exerts a synergistic regulatory role in bone defect repair (Liu et al., 2020; Zhang M. et al., 2022; Liao et al., 2024; Zeng et al., 2025). For instance, mitochondria derived from BMSCs can be transferred to functionally impaired osteoblasts via exosomes, restoring their oxidative phosphorylation activity and mineralization capacity. Meanwhile, mitochondria released by osteoblasts can be internalized by osteoclasts, and inhibit excessive bone resorption through modulating the intracellular ROS levels. Such directional intercellular mitochondrial trafficking constructs a functional synergy network among distinct cell populations within the bone repair microenvironment, thereby providing an efficient regulatory pathway for bone regeneration. Additionally, mitochondrial transfer between neurons and BMSCs can enhance the nerve-bone crosstalk, which further improves the bone regeneration efficiency of critical-sized defects. These findings collectively highlight the crucial role of mitochondrial trafficking in multi-system coordinated bone repair.

Extensive research confirms that imbalances in mitochondrial dynamics—encompassing fusion, fission, autophagy, and transport—constitute a critical pathological link in numerous bone-related disorders. By precisely regulating energy metabolism, oxidative stress levels, and signalling pathway activation within bone cells, mitochondrial dynamics directly influences the functional activity of core cells such as osteoblasts, osteoclasts, and BMSCs, thereby determining the efficiency of bone defect repair. Current research indicates that mitochondrial-targeting biomaterials offer novel strategies for bone defect repair by restoring mitochondrial morphology and function while promoting osteogenic differentiation. However, the precise molecular mechanisms by which mitochondrial dynamics processes—particularly the coordination of fusion and fission, and the coupling of transport and autophagy—regulate osteogenic differentiation remain unclear and warrant further investigation.

## 4 Synergistic role of the neuro-vascular-muscular axis and mitochondrial regulation in bone defect repair

Bone defect repair constitutes a complex pathophysiological process involving multiple systems, wherein the nervous, vascular, and muscular systems form a synergistic network through their respective functional characteristics to jointly regulate the bone regeneration process. Mitochondria, serving as the core regulatory hub of cellular function, participate in the coordinated regulation of the neuro-vascular-muscular axis through multidimensional actions, thereby facilitating bone defect repair: Firstly, by maintaining mitochondrial homeostasis and energy supply in neurons, ensuring normal transmission of neural signals; Secondly, by enhancing oxidative phosphorylation activity in vascular endothelial cells and regulating VEGF secretion to promote angiogenesis; Thirdly, by improving mitochondrial function in myogenic stem cells, thereby enhancing their proliferation and differentiation potential. These mechanisms provide crucial theoretical foundations for mitochondrial-targeted adjuvant therapies in bone defect repair.

### 4.1 Neurons

As a highly innervated organ, bone harbours intricate functional interactions between its intrinsic nerve fibres and skeletal cells such as osteoblasts and osteoclasts (Sun W. et al., 2023). Neural tissue maintains skeletal homeostasis through multidimensional mechanisms: regulating local blood flow within bone tissue, precisely modulating bone metabolic equilibrium, secreting neurotransmitters and neuropeptides, and controlling the proliferation and differentiation of stem cells including BMSCs (Zhang Z. et al., 2022). During the early stages of bone defect repair, nerve regeneration frequently initiates prior to bone regeneration, rendering the bone repair process markedly neuro-dependent. Chen et al. (2019) demonstrated that knocking out the prostaglandin E2 receptor 4 (EP4) gene in sensory nerves resulted in significantly reduced bone mass and markedly diminished osteoblast numbers in adult mice. Furthermore, research by Xia et al. (2021) demonstrated that EVs released from hippocampal neurons following traumatic brain injury can carry miR-328a-3p and miR-150-5p to target bone progenitor cells, directly stimulating osteogenic pathways and promoting bone formation. These studies indicate that neurogenic factors can regulate bone regeneration and maintain bone homeostasis by targeting distinct bone metabolism-related cells or organs.

Nerve growth factor (NGF) and its receptor p75 constitute one of the core regulatory pathways governing bone formation and metabolism. This signalling pathway promotes osteocyte migration, proliferation, and differentiation by activating downstream signalling molecules. Deficiency in NGF-p75 signalling leads to impaired sensory innervation of bone tissue, delayed local vascularisation, reduced numbers of bone progenitor cells, and skeletal developmental disorders (Xu et al., 2022), ultimately potentially affecting bone healing. Moreover, neuropeptides such as calcitonin gene-related peptide (CGRP), substance P (SP), vasoactive intestinal peptide (VIP), and neuropeptide Y (NPY) participate in bone formation and metabolism by binding to specific receptors on osteocyte surfaces (Sun W. et al., 2023). Additionally, these neuropeptides indirectly promote bone regeneration by regulating angiogenesis and the activity of immune cells.

The morphogenesis, functional maintenance, and repair of neuronal damage are highly dependent on mitochondrial function. Mitochondria safeguard neural function through the following mechanisms, thereby indirectly supporting the neurodependence of bone repair. Mitochondria dynamically adjust their morphology and activity according to the spatial and temporal demands of neurons, providing both energy and structural support for neuronal function. Established research confirms that mitochondrial dysfunction constitutes a significant precipitating factor in neurodegenerative diseases. Mitochondrial homeostasis and energy metabolism represent pivotal regulatory elements in damaged neuronal repair, serving as core regulatory hubs for neuronal responses to injury (Garone et al., 2024). Furthermore, maintaining normal Ca^2+^ levels is essential for proper neural signal transmission (Rosenberg and Spitzer, 2011). Mitochondria regulate Ca^2+^ concentrations between the cytoplasm and mitochondrial matrix through mechanisms such as the mitochondrial calcium uncoupling protein (MCU), preventing neuronal damage from abnormal Ca^2+^ accumulation and ensuring stable neural signal transmission. The directed transport of mitochondria within neuronal axons provides the energy foundation for axonal regeneration. Damaged neurons enhance mitochondrial mobility towards injured axonal sites, supplying ample ATP to facilitate axonal regrowth and promote functional recovery of injured neurons (Zhou et al., 2016).

### 4.2 Blood vessels

Blood vessels are extensively distributed throughout bone tissue. The growth of blood vessels and the establishment of vascular networks facilitate the transport of oxygen, nutrients, and immune cells to sites of bone defects, forming a prerequisite for bone defect repair (Sadeghsoltani et al., 2024). As a complex process involving multicellular cooperation, angiogenesis is closely linked to mitochondrial function. Mitochondria not only supply energy to endothelial cells but also regulate their metabolism through associated proteins and signalling molecules (Luo et al., 2023). Furthermore, ROS signalling molecules produced by mitochondria stabilise HIF-1α, thereby inducing VEGF production (Reichard and Asosingh, 2019). This subsequently promotes endothelial cell migration, proliferation, and angiogenesis, ultimately influencing the efficiency of vascularisation and the outcome of bone defect repair.

TCV constitute a distinct category of capillaries originating from the bone marrow and composed of endothelial cells spanning the cortical bone (Grüneboom et al., 2019). By connecting the periosteum and periosteum, they provide a crucial foundation for cellular communication and material exchange. As demonstrated by Liao et al. (2024), osteoblasts maintain the structural and functional homeostasis of the TCV network by transferring mitochondria to cortical bone endothelial cells. Further mechanistic studies suggest that osteocyte-derived mitochondria may regulate endothelial proliferation by enhancing the sphingosine signalling pathway. Collectively, osteocyte-to-endothelial mitochondrial transfer maintains endothelial function through multiple pathways: mitigating oxidative stress damage, promoting endothelial proliferation and migration, and enhancing vasculogenesis capacity. This ultimately supports bone regeneration by sustaining TCV homeostasis. Wang et al. (2024a) isolated mitochondria from BMSCs and transplanted them into BMSCs in a co-culture system, successfully establishing a “BMSCs-mitochondria (BMSCs-mito) system”. Results demonstrated that the transplanted system significantly promoted functional vascular network formation and accelerated bone defect healing. Mechanistically, mitochondrial transplantation was found to enhance vascular endothelial cell synergy by activating the DLL4-Notch1 signalling pathway, offering a novel strategy for bone repair. Further corroborating the broad restorative value of mitochondrial transplantation, research by Levoux et al. (2021) demonstrated its efficacy not only in promoting wound healing and muscle repair but also in enhancing the angiogenic potential of endothelial cells under ischaemic conditions. This provides theoretical support for treating ischaemic bone defects, such as diabetic non-union. Collectively, these findings indicate that mitochondrial transplantation, as an emerging therapeutic approach, holds considerable promise for application in the field of bone repair.

### 4.3 Muscle

The functional synergy between bone and muscle extends beyond mechanical coupling to form a profoundly interconnected regulatory network via molecular signalling pathways. As the core hub for musculoskeletal coordinated repair, mitochondria maintain muscle energy metabolism and the secretion of myokines. Once mitochondrial function is impaired, it leads to a reduction in muscle ATP production, a decrease in myokines levels, and a decline in osteogenic markers.

As a functional endocrine organ, muscle secretes multiple myokines (Table 2), which modulate bone metabolic homeostasis by targeting bone tissue cells (Kim, 2022). Concurrently, muscle-derived stem cells (MDSCs), serving as a primary source of bone progenitor cells, can differentiate directionally into chondrocytes and osteoblasts, directly participating in bone defect repair processes (Shah et al., 2013). However, in cases of fractures accompanied by extensive muscle injury, bone defect repair remains suboptimal even with intervention by osteogenic regulators such as BMP-2, frequently inducing complications that lead to poor prognosis (Borok et al., 2020). Moreover, with advancing age or disease impact, mitochondrial autophagy function within skeletal muscle may become impaired, leading to the accumulation of dysfunctional mitochondria. This not only compromises muscle contraction and secretory functions but also indirectly disrupts the microenvironment for bone defect repair.

**TABLE 2:** Regulation of bone metabolism by myokines: mechanisms and contexts.

Myokine	Primary inducers of secretion	Molecular mechanisms in osteoblasts
Irisin	Exercise	Activates integrin receptors (e.g., αV/β5), leading to the activation of the canonical Wnt/β-catenin pathway, promoting osteogenic differentiation. Concurrently inhibits osteoclastogenesis
IGF-1	Exercise, mechanical loading	Binds to IGF-1R, activating PI3K/Akt/mTOR and MAPK/ERK pathways to stimulate osteoblast proliferation, differentiation, survival, and bone matrix synthesis
BDNF	Exercise	Signals through the TrkB receptor, activating PI3K/Akt and MAPK/ERK pathways to promote osteoblast differentiation, mineralization, and cell survival
IL-11	Muscle contraction	Signals through the GP130/JAK/STAT3 pathway to upregulate osteogenic master regulators (e.g., *Runx2*)
FGF-2 (bFGF)	Exercise, muscle injury	Binds to FGFR1, primarily stimulating the MAPK/ERK pathway to induce potent proliferation of osteoprogenitor cells, crucial for bone repair
Myostatin (GDF-8)	Muscle atrophy, disuse, aging	Signals through ActRIIB, inducing Smad2/3 phosphorylation to transcriptionally repress osteogenic genes (e.g., *Runx2*). Promotes RANKL expression
TNF-α	Chronic, Inflammation, aging	Activates NF-κB signaling, which inhibits the Wnt/β-catenin pathway. Induces osteoblast apoptosis and stimulates osteoclastogenesis via RANKL.
IL-6	Acute: Exercise Chronic: Inflammation, aging	Acute: Mild anabolic effects via MAPK/ERK. Chronic: Sustained signaling via JAK/STAT3 and NF-κB stimulates RANKL production, promoting osteoclastogenesis
FGF-21	Exercise, Fasting, metabolic stress	Physiological: Metabolic regulation. Pathological: Chronic elevation increases RANKL/OPG ratio and may inhibit differentiation (e.g., *Osterix* suppression)

## 5 Outlook, perspective, and conclusion

There exists an intricate relationship between mitochondria and bone defect repair. This paper systematically examines the mechanisms by which mitochondria influence bone defect repair, addressing three key aspects: mitochondrial function, mitochondrial dynamics, and the interplay between mitochondria and tissues. Mitochondria promote bone tissue repair by regulating cells (such as bone marrow mesenchymal cells, osteoblasts, and osteoclasts), nerves, muscles, and angiogenesis. Increasing evidence indicates that mitochondrial structural and functional impairments constitute key factors in disorders such as impaired bone repair, osteoporosis, osteoarthritis, and osteogenesis imperfecta. Consequently, mitochondrial-targeted therapeutic strategies have emerged. By restoring mitochondrial structure and function, these approaches regulate bone homeostasis, laying the groundwork for clinical interventions in bone defect repair. Nevertheless, the precise molecular mechanisms underpinning mitochondrial promotion of bone repair remain incompletely understood, with relatively limited research in this area. Deepening our understanding of the relationship between mitochondria and bone repair will not only shed light on the intrinsic mechanisms of bone defect repair but also provide crucial evidence for developing novel therapeutic drugs and treatment approaches. Future research should focus on the signalling pathways and metabolic regulatory mechanisms of mitochondria in bone repair, aiming to deliver safer and more effective solutions for clinical treatment.

## Glossary

ATP: Adenosine Triphosphate
ADP: Adenosine Diphosphate
OXPHOS: Oxidative Phosphorylation
ROS: Reactive Oxygen Species
BMSCs: Bone Marrow Mesenchymal Stem Cells
**NAD** ^**+**^: Nicotinamide Adenine Dinucleotide
NADH: Nicotinamide Adenine Dinucleotide
**FADH**_**2**_: Flavin Adenine Dinucleotide
TAC: Tricarboxylic Acid Cycle
ΔΨ: Mitochondrial Membrane Potential
Apaf-1: Apoptotic Protease Activating Factor 1
Caspase Family: Cysteine Aspartate Specific Protease Family
BAX: BCL2-Associated X Protein
VDAC: Voltage-Dependent Anion Channel
Hsp60: Heat Shock Protein 60
p38: p38 Mitogen-Activated Protein Kinase
MK2: MAPK-Activated Protein Kinase 2
IKKα/IKKγ/IKKβ: IκB Kinase α/γ/β
IκB: Inhibitor of NF-κB
NF-κB: Nuclear Factor Kappa-Light-Chain-Enhancer of Activated B Cells
Cyt C: Cytochrome C
Bcl-2: B-Cell Lymphoma 2
GPX4: Glutathione Peroxidase 4
RLR: RIG-I-Like Receptors
MAVS: Mitochondrial Antiviral Signaling Protein
TRAF: Tumor Necrosis Factor Receptor-Associated Factor
IRF-3: Interferon Regulatory Factor 3
mtDNA: Mitochondrial DNA
TLR9: Toll-Like Receptor 9
NLRP3: NOD-Like Receptor Pyrin Domain-Containing Protein 3
GSDMD: Gasdermin D
IFN-α/β: Interferon α/β
IL-1β/18: Interleukin-1β/18
TNF-α: Tumor Necrosis Factor α
DRP1: Dynamin-Related Protein 1
MFN1/2: Mitofusin 1/2
FIS1: Mitochondrial Fission 1 Protein
OPA1: Optic Atrophy 1

## References

[B1] Altieri D. C. (2019). Mitochondrial dynamics and metastasis. Cell. Mol. Life Sci. 76, 827–835. 10.1007/s00018-018-2961-2 30415375 PMC6559795

[B2] An Y. Liu W. J. Xue P. Ma Y. Zhang L. Q. Zhu B. (2018). Autophagy promotes MSC-mediated vascularization in cutaneous wound healing *via* regulation of VEGF secretion. Cell Death Dis. 9, 58. 10.1038/s41419-017-0082-8 29352190 PMC5833357

[B3] Antony A. N. Paillard M. Moffat C. Juskeviciute E. Correnti J. Bolon B. (2016). MICU1 regulation of mitochondrial Ca2+ uptake dictates survival and tissue regeneration. Nat. Commun. 7, 10955. 10.1038/ncomms10955 26956930 PMC4786880

[B4] Ballard A. Zeng R. Zarei A. Shao C. Cox L. Yan H. (2020). The tethering function of mitofusin2 controls osteoclast differentiation by modulating the Ca2+–NFATc1 axis. J. Biol. Chem. 295, 6629–6640. 10.1074/jbc.RA119.012023 32165499 PMC7212632

[B5] Bao J. Wei Y. Chen L. (2024). Research progress on the regulatory cell death of osteoblasts in periodontitis. J. Zhejiang Univ. Med. Sci. 53, 533–540. 10.3724/zdxbyxb-2024-0038 38803282 PMC11528140

[B6] Bhatti J. S. Bhatti G. K. Reddy P. H. (2017). Mitochondrial dysfunction and oxidative stress in metabolic disorders — a step towards mitochondria based therapeutic strategies. Biochimica Biophysica Acta (BBA) - Mol. Basis Dis. 1863, 1066–1077. 10.1016/j.bbadis.2016.11.010 27836629 PMC5423868

[B7] Borok M. J. Mademtzoglou D. Relaix F. (2020). Bu-M-P-ing iron: how BMP signaling regulates muscle growth and regeneration. JDB 8, 4. 10.3390/jdb8010004 32053985 PMC7151139

[B8] Borriello A. Caldarelli I. Speranza M. C. Scianguetta S. Tramontano A. Bencivenga D. (2016). Iron overload enhances human mesenchymal stromal cell growth and hampers matrix calcification. Biochimica Biophysica Acta (BBA) - General Subj. 1860, 1211–1223. 10.1016/j.bbagen.2016.01.025 26850692

[B9] Brestoff J. R. Singh K. K. Aquilano K. Becker L. B. Berridge M. V. Boilard E. (2025). Recommendations for mitochondria transfer and transplantation nomenclature and characterization. Nat. Metab. 7, 53–67. 10.1038/s42255-024-01200-x 39820558

[B10] Castro I. G. Richards D. M. Metz J. Costello J. L. Passmore J. B. Schrader T. A. (2018). A role for mitochondrial rho GTPase 1 (MIRO1) in motility and membrane dynamics of peroxisomes. Traffic 19, 229–242. 10.1111/tra.12549 29364559 PMC5888202

[B11] Ceccariglia S. Cargnoni A. Silini A. R. Parolini O. (2020). Autophagy: a potential key contributor to the therapeutic action of mesenchymal stem cells. Autophagy 16, 28–37. 10.1080/15548627.2019.1630223 31185790 PMC6984485

[B12] Chan D. C. (2020). Mitochondrial dynamics and its involvement in disease. Annu. Rev. Pathol. Mech. Dis. 15, 235–259. 10.1146/annurev-pathmechdis-012419-032711 31585519

[B13] Che J. Lv H. Yang J. Zhao B. Zhou S. Yu T. (2021). Iron overload induces apoptosis of osteoblast cells *via* eliciting ER stress-mediated mitochondrial dysfunction and p-eIF2α/ATF4/CHOP pathway *in vitro* . Cell. Signal. 84, 110024. 10.1016/j.cellsig.2021.110024 33901579

[B14] Chen H. Hu B. Lv X. Zhu S. Zhen G. Wan M. (2019). Prostaglandin E2 mediates sensory nerve regulation of bone homeostasis. Nat. Commun. 10, 181. 10.1038/s41467-018-08097-7 30643142 PMC6331599

[B15] Chen W. R. Zhou Y. J. Sha Y. Wu X. P. Yang J. Q. Liu F. (2020). Melatonin attenuates vascular calcification by inhibiting mitochondria fission *via* an AMPK/Drp1 signalling pathway. J. Cell. Mol. Medi 24, 6043–6054. 10.1111/jcmm.15157 32368857 PMC7294128

[B16] Chen X. He Q. Zhai Q. Tang H. Li D. Zhu X. (2023a). Adaptive nanoparticle-mediated modulation of mitochondrial homeostasis and inflammation to enhance infected bone defect healing. ACS Nano 17, 22960–22978. 10.1021/acsnano.3c08165 37930276

[B17] Chen Y. Fang Z.-M. Yi X. Wei X. Jiang D.-S. (2023b). The interaction between ferroptosis and inflammatory signaling pathways. Cell Death Dis. 14, 205. 10.1038/s41419-023-05716-0 36944609 PMC10030804

[B18] Chen S. Li Q. Shi H. Li F. Duan Y. Guo Q. (2024). New insights into the role of mitochondrial dynamics in oxidative stress-induced diseases. Biomed. and Pharmacother. 178, 117084. 10.1016/j.biopha.2024.117084 39088967

[B19] Clarke B. (2008). Normal bone anatomy and physiology. Clin. J. Am. Soc. Nephrol. 3, S131–S139. 10.2215/CJN.04151206 18988698 PMC3152283

[B20] Ding Y. Yang H. Gao J. Tang C. Peng Y.-Y. Ma X.-M. (2025). Synaptic-mitochondrial transport: mechanisms in neural adaptation and degeneration. Mol. Cell Biochem. 480, 3399–3411. 10.1007/s11010-025-05209-y 39841406

[B21] Dobson P. F. Dennis E. P. Hipps D. Reeve A. Laude A. Bradshaw C. (2020). Mitochondrial dysfunction impairs osteogenesis, increases osteoclast activity, and accelerates age related bone loss. Sci. Rep. 10, 11643. 10.1038/s41598-020-68566-2 32669663 PMC7363892

[B22] Dorn G. W. (2019). Evolving concepts of mitochondrial dynamics. Annu. Rev. Physiol. 81, 1–17. 10.1146/annurev-physiol-020518-114358 30256725

[B23] D’Angelo D. Rizzuto R. (2023). The mitochondrial calcium uniporter (MCU): molecular identity and role in human diseases. Biomolecules 13, 1304. 10.3390/biom13091304 37759703 PMC10526485

[B24] Fan L. Wang J. Ma C. (2020). miR125a attenuates BMSCs apoptosis *via* the MAPK‐ERK pathways in the setting of craniofacial defect reconstruction. J. Cell. Physiology 235, 2857–2865. 10.1002/jcp.29191 31578723

[B25] Forni M. F. Peloggia J. Trudeau K. Shirihai O. Kowaltowski A. J. (2016). Murine mesenchymal stem cell commitment to differentiation is regulated by mitochondrial dynamics. Stem Cells 34, 743–755. 10.1002/stem.2248 26638184 PMC4803524

[B26] Garone C. De Giorgio F. Carli S. (2024). Mitochondrial metabolism in neural stem cells and implications for neurodevelopmental and neurodegenerative diseases. J. Transl. Med. 22, 238. 10.1186/s12967-024-05041-w 38438847 PMC10910780

[B27] Ghosh S. Sinha M. Samanta R. Sadhasivam S. Bhattacharyya A. Nandy A. (2022). A potent antibiotic-loaded bone-cement implant against staphylococcal bone infections. Nat. Biomed. Eng. 6, 1180–1195. 10.1038/s41551-022-00950-x 36229662 PMC10101771

[B28] Glover H. L. Schreiner A. Dewson G. Tait S. W. G. (2024). Mitochondria and cell death. Nat. Cell Biol. 26, 1434–1446. 10.1038/s41556-024-01429-4 38902422

[B29] Grüneboom A. Hawwari I. Weidner D. Culemann S. Müller S. Henneberg S. (2019). A network of trans-cortical capillaries as mainstay for blood circulation in long bones. Nat. Metab. 1, 236–250. 10.1038/s42255-018-0016-5 31620676 PMC6795552

[B30] Guo Y. Chi X. Wang Y. Heng B. C. Wei Y. Zhang X. (2020). Mitochondria transfer enhances proliferation, migration, and osteogenic differentiation of bone marrow mesenchymal stem cell and promotes bone defect healing. Stem Cell Res. Ther. 11, 245. 10.1186/s13287-020-01704-9 32586355 PMC7318752

[B31] Guo Z. Wu J. Hu Y. Zhou J. Li Q. Zhang Y. (2024). Exogenous iron caused osteocyte apoptosis, increased RANKL production, and stimulated bone resorption through oxidative stress in a murine model. Chemico-Biological Interact. 399, 111135. 10.1016/j.cbi.2024.111135 38971422

[B32] Han Y. Li X. Zhang Y. Han Y. Chang F. Ding J. (2019). Mesenchymal stem cells for regenerative medicine. Cells 8, 886. 10.3390/cells8080886 31412678 PMC6721852

[B33] He Q. Zhao Q. Li Q. Pan R. Li X. Chen Y. (2021). Mtu1 defects are correlated with reduced osteogenic differentiation. Cell Death Dis. 12, 61. 10.1038/s41419-020-03345-5 33431792 PMC7801634

[B34] Hsu Y.-C. Wu Y.-T. Yu T.-H. Wei Y.-H. (2016). Mitochondria in mesenchymal stem cell biology and cell therapy: from cellular differentiation to mitochondrial transfer. Semin. Cell Dev. Biol. 52, 119–131. 10.1016/j.semcdb.2016.02.011 26868759

[B35] Huo Y. Sun W. Shi T. Gao S. Zhuang M. (2022). The MFN1 and MFN2 mitofusins promote clustering between mitochondria and peroxisomes. Commun. Biol. 5, 423. 10.1038/s42003-022-03377-x 35523862 PMC9076876

[B36] Ighodaro O. M. Akinloye O. A. (2018). First line defence antioxidants-superoxide dismutase (SOD), catalase (CAT) and glutathione peroxidase (GPX): their fundamental role in the entire antioxidant defence grid. Alexandria J. Med. 54, 287–293. 10.1016/j.ajme.2017.09.001

[B37] Khalid S. Yamazaki H. Socorro M. Monier D. Beniash E. Napierala D. (2020). Reactive oxygen species (ROS) generation as an underlying mechanism of inorganic phosphate (Pi)-induced mineralization of osteogenic cells. Free Radic. Biol. Med. 153, 103–111. 10.1016/j.freeradbiomed.2020.04.008 32330587 PMC7262875

[B38] Kim B.-J. (2022). Effects of muscles on bone Metabolism—With a focus on myokines. Ann. Geriatr. Med. Res. 26, 63–71. 10.4235/agmr.22.0054 35722780 PMC9271391

[B39] Kim J.-M. Lin C. Stavre Z. Greenblatt M. B. Shim J.-H. (2020). Osteoblast-osteoclast communication and bone homeostasis. Cells 9, 2073. 10.3390/cells9092073 32927921 PMC7564526

[B40] Levoux J. Prola A. Lafuste P. Gervais M. Chevallier N. Koumaiha Z. (2021). Platelets facilitate the wound-healing capability of mesenchymal stem cells by mitochondrial transfer and metabolic reprogramming. Cell Metab. 33, 283–299.e9. 10.1016/j.cmet.2020.12.006 33400911

[B41] Li Q. Gao Z. Chen Y. Guan M.-X. (2017). The role of mitochondria in osteogenic, adipogenic and chondrogenic differentiation of mesenchymal stem cells. Protein Cell 8, 439–445. 10.1007/s13238-017-0385-7 28271444 PMC5445026

[B42] Li H. Zhang S. Huo S. Tang H. Nie B. Qu X. (2020). Effects of staphylococcal infection and aseptic inflammation on bone mass and biomechanical properties in a rabbit model. J. Orthop. Transl. 21, 66–72. 10.1016/j.jot.2019.11.006 32099806 PMC7029375

[B43] Li B. Shi Y. Liu M. Wu F. Hu X. Yu F. (2022). Attenuates of NAD+ impair BMSC osteogenesis and fracture repair through OXPHOS. Stem Cell Res. Ther. 13, 77. 10.1186/s13287-022-02748-9 35193674 PMC8864833

[B44] Li W. Wu Y. Zhang X. Wu T. Huang K. Wang B. (2023). Self-healing hydrogels for bone defect repair. RSC Adv. 13, 16773–16788. 10.1039/d3ra01700a 37283866 PMC10240173

[B45] Li Y. Fu G. Gong Y. Li B. Li W. Liu D. (2022). BMP-2 promotes osteogenic differentiation of mesenchymal stem cells by enhancing mitochondrial activity. J. Musculoskelet. Neuronal. Interact. 2, 123–131. 35234167 PMC8919656

[B46] Liao P. Chen L. Zhou H. Mei J. Chen Z. Wang B. (2024). Osteocyte mitochondria regulate angiogenesis of transcortical vessels. Nat. Commun. 15, 2529. 10.1038/s41467-024-46095-0 38514612 PMC10957947

[B47] Liu W. Li L. Rong Y. Qian D. Chen J. Zhou Z. (2020). Hypoxic mesenchymal stem cell-derived exosomes promote bone fracture healing by the transfer of miR-126. Acta Biomater. 103, 196–212. 10.1016/j.actbio.2019.12.020 31857259

[B48] Liu Z. Wang T. Zhang L. Luo Y. Zhao J. Chen Y. (2024). Metal–phenolic networks‐reinforced extracellular matrix scaffold for bone regeneration *via* combining radical‐scavenging and photo‐responsive regulation of microenvironment. Adv. Healthc. Mater. 13, 2304158. 10.1002/adhm.202304158 38319101

[B49] Luo Z. Yao J. Wang Z. Xu J. (2023). Mitochondria in endothelial cells angiogenesis and function: current understanding and future perspectives. J. Transl. Med. 21, 441. 10.1186/s12967-023-04286-1 37407961 PMC10324193

[B50] Ma L. Cheng Y. Feng X. Zhang X. Lei J. Wang H. (2024). A Janus‐ROS healing system promoting infectious bone regeneration *via* sono‐epigenetic modulation. Adv. Mater. 36, 2307846. 10.1002/adma.202307846 37855420

[B51] Mancuso E. Shah L. Jindal S. Serenelli C. Tsikriteas Z. M. Khanbareh H. (2021). Additively manufactured BaTiO3 composite scaffolds: a novel strategy for load bearing bone tissue engineering applications. Mater Sci. Eng. C Mater Biol. Appl. 126, 112192. 10.1016/j.msec.2021.112192 34082989

[B52] Mills E. L. Kelly B. O’Neill L. A. J. (2017). Mitochondria are the powerhouses of immunity. Nat. Immunol. 18, 488–498. 10.1038/ni.3704 28418387

[B53] Mishra P. Chan D. C. (2016). Metabolic regulation of mitochondrial dynamics. J. Cell Biol. 212, 379–387. 10.1083/jcb.201511036 26858267 PMC4754720

[B54] Modesti L. Danese A. Angela Maria Vitto V. Ramaccini D. Aguiari G. Gafà R. (2021). Mitochondrial Ca2+ signaling in health, disease and therapy. Cells 10, 1317. 10.3390/cells10061317 34070562 PMC8230075

[B55] Morse P. T. Arroum T. Wan J. Pham L. Vaishnav A. Bell J. (2024). Phosphorylations and acetylations of cytochrome c control mitochondrial respiration, mitochondrial membrane potential, energy, ROS, and apoptosis. Cells 13, 493. 10.3390/cells13060493 38534337 PMC10969761

[B56] Pays E. (2025). Inflammation-linked apolipoprotein-L activities: immunity control, mitochondrial repair, pathogen resistance, and disease induction. Regen. Med. Rep. 2, 161–168. 10.4103/REGENMED.REGENMED-D-25-00027

[B57] Pernas L. Scorrano L. (2016). Mito-morphosis: Mitochondrial fusion, fission, and cristae remodeling as key mediators of cellular function. Annu. Rev. Physiol. 78, 505–531. 10.1146/annurev-physiol-021115-105011 26667075

[B58] Qiu X. Wang X. Qiu J. Zhu Y. Liang T. Gao B. (2019). Melatonin rescued reactive oxygen species-impaired osteogenesis of human bone marrow mesenchymal stem cells in the presence of tumor necrosis factor-alpha. Stem Cells Int. 2019, 1–11. 10.1155/2019/6403967 31582985 PMC6754961

[B59] Quintana-Cabrera R. Scorrano L. (2023). Determinants and outcomes of mitochondrial dynamics. Mol. Cell 83, 857–876. 10.1016/j.molcel.2023.02.012 36889315

[B60] Reichard A. Asosingh K. (2019). The role of mitochondria in angiogenesis. Mol. Biol. Rep. 46, 1393–1400. 10.1007/s11033-018-4488-x 30460535 PMC6426673

[B61] Ren L. Chen X. Chen X. Li J. Cheng B. Xia J. (2020). Mitochondrial dynamics: fission and fusion in fate determination of mesenchymal stem cells. Front. Cell Dev. Biol. 8, 580070. 10.3389/fcell.2020.580070 33178694 PMC7593605

[B62] Rigoulet M. Bouchez C. L. Paumard P. Ransac S. Cuvellier S. Duvezin-Caubet S. (2020). Cell energy metabolism: an update. Biochimica Biophysica Acta (BBA) - Bioenergetics 1861, 148276. 10.1016/j.bbabio.2020.148276 32717222

[B63] Rogers M. A. Maldonado N. Hutcheson J. D. Goettsch C. Goto S. Yamada I. (2017). Dynamin-related protein 1 inhibition attenuates cardiovascular calcification in the presence of oxidative stress. Circulation Res. 121, 220–233. 10.1161/CIRCRESAHA.116.310293 28607103 PMC5546003

[B64] Rosenberg S. S. Spitzer N. C. (2011). Calcium signaling in neuronal development. Cold Spring Harb. Perspect. Biol. 3, a004259. 10.1101/cshperspect.a004259 21730044 PMC3179332

[B65] Sadeghsoltani F. Hassanpour P. Safari M. Haiaty S. Rahbarghazi R. Rahmati M. (2024). Angiogenic activity of mitochondria; beyond the sole bioenergetic organelle. J. Cell. Physiology 239, e31185. 10.1002/jcp.31185 38219050

[B66] Sart S. Song L. Li Y. (2015). Controlling redox status for stem cell survival, expansion, and differentiation. Oxid. Med. Cell Longev. 2015, 105135. 10.1155/2015/105135 26273419 PMC4530287

[B67] Saunders T. L. Windley S. P. Gervinskas G. Balka K. R. Rowe C. Lane R. (2024). Exposure of the inner mitochondrial membrane triggers apoptotic mitophagy. Cell Death Differ. 31, 335–347. 10.1038/s41418-024-01260-2 38396150 PMC10923902

[B68] Shah K. Majeed Z. Jonason J. O’Keefe R. J. (2013). The role of muscle in bone repair: the cells, signals, and tissue responses to injury. Curr. Osteoporos. Rep. 11, 130–135. 10.1007/s11914-013-0146-3 23591779 PMC3698863

[B69] Sheehy E. J. Kelly D. J. O’Brien F. J. (2019). Biomaterial-based endochondral bone regeneration: a shift from traditional tissue engineering paradigms to developmentally inspired strategies. Mater Today Bio 3, 100009. 10.1016/j.mtbio.2019.100009 32159148 PMC7061547

[B70] Shuai C. Zan J. Deng F. Yang Y. Peng S. Zhao Z. (2021). Core–shell-Structured ZIF-8@PDA-HA with controllable zinc ion release and superior bioactivity for improving a Poly-l -lactic acid scaffold. ACS Sustain. Chem. Eng. 9, 1814–1825. 10.1021/acssuschemeng.0c08009

[B71] Sun H. Xu J. Wang Y. Shen S. Xu X. Zhang L. (2023a). Bone microenvironment regulative hydrogels with ROS scavenging and prolonged oxygen-generating for enhancing bone repair. Bioact. Mater. 24, 477–496. 10.1016/j.bioactmat.2022.12.021 36714330 PMC9843284

[B72] Sun W. Ye B. Chen S. Zeng L. Lu H. Wan Y. (2023b). Neuro–bone tissue engineering: emerging mechanisms, potential strategies, and current challenges. Bone Res. 11, 65. 10.1038/s41413-023-00302-8 38123549 PMC10733346

[B73] Tang D. Chen X. Kang R. Kroemer G. (2021). Ferroptosis: molecular mechanisms and health implications. Cell Res. 31, 107–125. 10.1038/s41422-020-00441-1 33268902 PMC8026611

[B74] Tao H. Ge G. Liang X. Zhang W. Sun H. Li M. (2020). ROS signaling cascades: dual regulations for osteoclast and osteoblast. ABBS 52, 1055–1062. 10.1093/abbs/gmaa098 33085739

[B75] Tsang C. K. Liu Y. Thomas J. Zhang Y. Zheng X. F. S. (2014). Superoxide dismutase 1 acts as a nuclear transcription factor to regulate oxidative stress resistance. Nat. Commun. 5, 3446. 10.1038/ncomms4446 24647101 PMC4678626

[B76] Van Gastel N. Carmeliet G. (2021). Metabolic regulation of skeletal cell fate and function in physiology and disease. Nat. Metab. 3, 11–20. 10.1038/s42255-020-00321-3 33398192

[B77] Wang S. Deng Z. Ma Y. Jin J. Qi F. Li S. (2020). The role of autophagy and mitophagy in bone metabolic disorders. Int. J. Biol. Sci. 16, 2675–2691. 10.7150/ijbs.46627 32792864 PMC7415419

[B78] Wang J. Zhang Y. Cao J. Wang Y. Anwar N. Zhang Z. (2023). The role of autophagy in bone metabolism and clinical significance. Autophagy 19, 2409–2427. 10.1080/15548627.2023.2186112 36858962 PMC10392742

[B79] Wang Y. Li W. Guo Y. Huang Y. Guo Y. Song J. (2024a). Mitochondria transplantation to bone marrow stromal cells promotes angiogenesis during bone repair. Adv. Sci. 11, 2403201. 10.1002/advs.202403201 39137351 PMC11497025

[B80] Wang Y. Lin S. Chen L. Li M. Zhu Z. Zhuang Z. (2024b). Mitochondrial components transferred by MSC-derived exosomes promoted bone regeneration under high salt microenvironment *via* DRP1/Wnt signaling. Nano Res. 17, 8301–8315. 10.1007/s12274-024-6758-3

[B81] Xia W. Xie J. Cai Z. Liu X. Wen J. Cui Z.-K. (2021). Damaged brain accelerates bone healing by releasing small extracellular vesicles that target osteoprogenitors. Nat. Commun. 12, 6043. 10.1038/s41467-021-26302-y 34654817 PMC8519911

[B82] Xia Y. Ge G. Xiao H. Wu M. Wang T. Gu C. (2023). REPIN1 regulates iron metabolism and osteoblast apoptosis in osteoporosis. Cell Death Dis. 14, 631. 10.1038/s41419-023-06160-w 37749079 PMC10519990

[B83] Xu J. Li Z. Tower R. J. Negri S. Wang Y. Meyers C. A. (2022). NGF-p75 signaling coordinates skeletal cell migration during bone repair. Sci. Adv. 10.1126/sciadv.abl5716 35302859 PMC8932666

[B84] Yan H. Zou T. Tuo Q. Xu S. Li H. Belaidi A. A. (2021). Ferroptosis: mechanisms and links with diseases. Sig Transduct. Target Ther. 6, 49. 10.1038/s41392-020-00428-9 33536413 PMC7858612

[B85] Yang Y.-H. Li B. Zheng X.-F. Chen J.-W. Chen K. Jiang S.-D. (2014). Oxidative damage to osteoblasts can be alleviated by early autophagy through the endoplasmic reticulum stress pathway—Implications for the treatment of osteoporosis. Free Radic. Biol. Med. 77, 10–20. 10.1016/j.freeradbiomed.2014.08.028 25224042

[B86] Yang Y. Li T. Li Z. Liu N. Yan Y. Liu B. (2020). Role of mitophagy in cardiovascular disease. Aging Dis. 11, 419–437. 10.14336/AD.2019.0518 32257551 PMC7069452

[B87] Yue W. Chen Z. Liu H. Yan C. Chen M. Feng D. (2014). A small natural molecule promotes mitochondrial fusion through inhibition of the deubiquitinase USP30. Cell Res. 24, 482–496. 10.1038/cr.2014.20 24513856 PMC3975501

[B88] Zacharioudakis E. Gavathiotis E. (2023). Mitochondrial dynamics proteins as emerging drug targets. Trends Pharmacol. Sci. 44, 112–127. 10.1016/j.tips.2022.11.004 36496299 PMC9868082

[B89] Zan J. Shuai Y. Zhang J. Zhao J. Sun B. Yang L. (2023). Hyaluronic acid encapsulated silver metal organic framework for the construction of a slow-controlled bifunctional nanostructure: antibacterial and anti-inflammatory in intrauterine adhesion repair. Int. J. Biol. Macromol. 230, 123361. 10.1016/j.ijbiomac.2023.123361 36693610

[B90] Zeng F. Fu H. Liu Y. Xu Z. Zhou T. (2025). Application and mechanism of cell therapy technology in the repair of spinal cord injury: a narrative review. Adv. Technol. Neurosci. 2, 16–26. 10.4103/ATN.ATN-D-24-00008

[B91] Zhang Y. Huang L. Li M. Yang P. Cheng L. Yang H. (2025). Association between relative fat mass and lumbar bone mineral density in US adults: a cross-sectional study of the national health and nutrition examination survey 2011–2018. Regen. Med. Rep. 2, 143–148. 10.4103/REGENMED.REGENMED-D-25-00016

[B92] Zhang M. Li Y. Feng T. Li R. Wang Z. Zhang L. (2022). Bone engineering scaffolds with exosomes: a promising strategy for bone defects repair. Front. Bioeng. Biotechnol. 10, 920378. 10.3389/fbioe.2022.920378 35782499 PMC9240482

[B93] Zhang Z. Hao Z. Xian C. Fang Y. Cheng B. Wu J. (2022). Neuro-bone tissue engineering: multiple potential translational strategies between nerve and bone. Acta Biomater. 153, 1–12. 10.1016/j.actbio.2022.09.023 36116724

[B94] Zheng Y. Chen Z. She C. Lin Y. Hong Y. Shi L. (2020). Four-octyl itaconate activates Nrf2 cascade to protect osteoblasts from hydrogen peroxide-induced oxidative injury. Cell Death Dis. 11, 772. 10.1038/s41419-020-02987-9 32943614 PMC7499214

[B95] Zhong Z. Liang S. Sanchez-Lopez E. He F. Shalapour S. Lin X. (2018). New mitochondrial DNA synthesis enables NLRP3 inflammasome activation. Nature 560, 198–203. 10.1038/s41586-018-0372-z 30046112 PMC6329306

[B96] Zhou B. Yu P. Lin M.-Y. Sun T. Chen Y. Sheng Z.-H. (2016). Facilitation of axon regeneration by enhancing mitochondrial transport and rescuing energy deficits. J. Cell Biol. 214, 103–119. 10.1083/jcb.201605101 27268498 PMC4932375

[B97] Zuo C. Zhao X. Shi Y. Wu W. Zhang N. Xu J. (2018). TNF-α inhibits SATB2 expression and osteoblast differentiation through NF-κB and MAPK pathways. Oncotarget 9, 4833–4850. 10.18632/oncotarget.23373 29435145 PMC5797016

